# Effect of COVID-19 on the implementation of a multifaceted intervention to improve teamwork and quality for hospitalized patients: a qualitative interview study

**DOI:** 10.1186/s12913-022-08795-5

**Published:** 2022-11-19

**Authors:** Iva A. Terwilliger, Milisa Manojlovich, Julie K. Johnson, Mark V. Williams, Kevin J. O’Leary

**Affiliations:** 1 Center for Education in Health Sciences, Northwestern University Feinberg School of Medicine, 633 N St Clair St, Chicago, IL USA; 2 Department of Systems, Populations, and Leadership, University of Michigan School of Nursing, Ann Arbor, MI USA; 3 Department of Surgery, Northwestern University Feinberg School of Medicine, Chicago, IL USA; 4 Division of Hospital Medicine, Washington University School of Medicine, St. Louis, MO USA; 5 Division of Hospital Medicine, Northwestern University Feinberg School of Medicine, Chicago, IL USA

**Keywords:** Quality improvement, Implementation science, Qualitative research, Teamwork, COVID-19

## Abstract

**Background:**

Healthcare organizations made major adjustments to deliver care during the COVID pandemic, yet little is known about how these adjustments shaped ongoing quality and safety improvement efforts. We aimed to understand how COVID affected four U.S. hospitals’ prospective implementation efforts in an ongoing quality improvement initiative, the ***RE****designing ****S****yst****E****ms to Improve ****T****eamwork and Quality for Hospitalized Patients* (RESET) project, which implemented complementary interventions to redesign systems of care for medical patients.

**Methods:**

We conducted individual semi-structured interviews with 40 healthcare professionals to determine how COVID influenced RESET implementation. We used conventional qualitative content analysis to inductively code transcripts and identify themes in MAXQDA 2020.

**Results:**

We identified three overarching themes and nine sub-themes. The three themes were (1) COVID exacerbated existing problems and created new ones. (2) RESET and other quality improvement efforts were not the priority during the pandemic. (3) Fidelity of RESET implementation regressed.

**Conclusion:**

COVID had a profound impact on the implementation of a multifaceted intervention to improve quality and teamwork in four hospitals. Notably, COVID led to a diversion of attention and effort away from quality improvement efforts, like RESET, and sites varied in their ability to renew efforts over time. Our findings help explain how COVID adversely affected hospitals’ quality improvement efforts throughout the pandemic and support the need for research to identify elements important for fostering hospital resilience.

**Supplementary Information:**

The online version contains supplementary material available at 10.1186/s12913-022-08795-5.

## Background

The SARS-CoV-2/Covid-19 (further: COVID) pandemic caused unprecedented disruptions to the healthcare industry, forcing healthcare organizations to make rapid, major adjustments to care delivery. Initial adjustments included reduction in non-essential services, creation of respiratory isolation units, and redeployment of professionals from areas of low patient volume to areas with greater need. Early on, experts called for the use of quality improvement methods and implementation science to help healthcare organizations select strategies and manage the challenges posed by the pandemic [1–4]. Yet, the need for rapid, major changes in how healthcare was delivered also raised concern that healthcare organizations pulled resources away from traditional programs for quality and patient safety [5]. Increased rates of hospital acquired infection during the first year of the pandemic support this concern [6–8]. Furthermore, acknowledging the enormous strain the pandemic placed on hospitals and unpredictable effect on quality measure performance, the U.S. Center for Medicare and Medicare Services granted exceptions to reporting requirements early in the pandemic and recently proposed continued suppression of several measures in the Hospital Value Based Purchasing and Hospital Acquired Conditions Reduction Programs [9, 10]. Although much is known about adjustments made by healthcare organizations during the pandemic, little is known about how these modifications shaped hospitals’ ongoing, non-COVID related quality and safety improvement efforts. Characterizing the ways in which hospitals’ responses to COVID influenced their quality improvement efforts is important for understanding the decrease in quality performance measures and identifying strategies to ensure high performance in the face of extreme challenges.

With the advent of COVID, our research team noticed a change in implementation efforts at hospitals that were participating in the ***RE****designing ****S****yst****E****ms to Improve ****T****eamwork and Quality for Hospitalized Patients* (RESET) project. The RESET project is a multi-year study that aims to improve teamwork and safety for hospitalized patients at four hospitals through the use of mentor dyads to facilitate implementation of the **A**dvanced and **I**ntegrated **M**icro**S**ystems (AIMS) interventions. These complementary and mutually reinforcing interventions address common challenges in providing safe, effective, patient-centered care to hospitalized medical patients [11]. The AIMS interventions consist of (1) Unit-based Physician Teams, (2) Unit Nurse-Physician Co-leadership, (3) Enhanced Interprofessional Rounds, (4) Unit-level Performance Reports, and (5) Patient Engagement Activities. Sites began mentored implementation in Fall 2018, before the pandemic, creating an opportunity for us to leverage this natural experiment to understand how COVID affected four geographically diverse hospitals’ ongoing implementation efforts. In this study, we sought to understand how hospitals’ experience during the COVID pandemic shaped implementation of RESET.

## Methods

### Study design and setting

We conducted a qualitative study consisting of semi-structured interviews with participants at all four RESET hospitals. We selected the four hospitals, among 14 hospitals which had applied for the RESET project, based on their need (i.e., similar intervention had not already been implemented) and evidence of organizational commitment to the project. Two of four study hospitals were located in the Southeast U.S., one in the Midwest, and one in the West. All hospitals were nonprofit with between 200 and 350 beds. Two were non-teaching hospitals and two were teaching hospitals, though neither was a major affiliate of a medical school. An overview of the AIMS interventions is provided in Table 1. More details about the sites, the interventions, and larger study are described elsewhere [11, 12]. This manuscript adheres to the Consolidated Criteria for Reporting Qualitative Research (COREQ) checklist [13]. This study was approved by the Northwestern University Institutional Review Board (STU00213677).

**Table 1:** Advanced and Integrated Microsystems (AIMS) Interventions

Components	Description
Unit-based Physician Teams	Localization of physician to a minimum number of units on which they provide care
Unit Nurse-Physician Co-leadership	Collaborative model in which a nurse leader and physician leader are jointly responsible for quality improvement on their unit
Enhanced Interprofessional Rounds	Interprofessional rounds, redesigned with input from frontline professionals to optimize collaboration and patient engagement
Unit-level Performance Reports	Performance reports designed to give unit leaders and frontline professionals relevant, interpretable, actionable data
Patient Engagement Activities	Methods to continually inform and engage patients and families as partners in care

### Sampling and recruitment

We used a purposive sampling strategy [14]. We invited the physician and nurse leader for each RESET study site to participate in semi-structured interviews and, at the end of each interview, asked them to identify additional potential interviewees with varying roles for us to recruit via email. Sampling was purposive in that we aimed to interview project leaders and at least one frontline nurse, physician, and case manager at each site. Eligible participants were either healthcare professionals who participated in RESET, leaders who had oversight of unit(s) that implemented RESET, or those who were involved in RESET implementation such as administrators.

### Data collection

The one-time individual semi-structured interviews were conducted via Zoom between November 2020 and June 2021 and were audio recorded. Interviews were guided by a pre-tested interview guide and were 45–60 min in length. Verbal informed consent was obtained before the start of the interview. The interviews were conducted by IAT, a female PhD candidate and nurse with training and experience in qualitative methods, but who otherwise had no role in RESET. A portion of the interviews were also jointly conducted by KJO, a male physician researcher with experience in qualitative methods and the principal investigator of RESET. Both researchers are experts in quality improvement. The interview protocol was structured to ask participants (1) how their work changed during COVID, (2) how COVID affected implementation of RESET, and (3) how RESET affected their response to COVID. The full interview guide is displayed in Additional File 1. We collected demographic data on age and professional role. No field notes were taken.

### Data analysis

Interviews were transcribed by an independent, professional transcription service and imported into MAXQDA 2020, a software for qualitative analysis [15]. We used an inductive approach and conducted conventional content analysis [16, 17]. In our first cycle coding, all coders (IAT, KJO, MM, and JKJ) independently reviewed and coded the first eight transcripts and collectively built a codebook. IAT coded all remaining transcripts using the codebook, and KJO, MM, and JKJ each received a portion of the remaining transcripts, which they independently coded so that each transcript was coded by two individuals: IAT and either KJO, MM, or JKJ. The team compared coding, resolved disagreements through iterative discussion, and refined the codebook. Our second cycle coding used pattern coding to compare, synthesize, and map relationships between findings; and generate interpretive insights about the data. As part of our second coding cycle, we used tools such as memos and data displays as outlined in the work of Miles, Huberman and Saldaña [17]. The coding team subsequently presented analysis and sought feedback from the larger research team via a recurring monthly teleconference. We analyzed all interview transcripts. Both code saturation (codebook is stable) and meaning saturation (understanding of issue with no additional insights arising) were met [18].

### Rigor

We addressed qualitative research rigor in the research design by involving a diverse group of expert researchers in the design, data collection, analysis, and interpretation of the study (investigator triangulation) [19]; and completing member checking [20]. Participant member checking occurred during the final RESET study call in March 2022 (transcripts and data analysis were not returned to participants.) To enhance the transferability of the findings, we provided descriptions of the concept under study, characteristics of the participants, method of data collection, methods used to analyze the data, and samples of the participants’ quotes so that judgements about the degree of fit or similarity may be made by others who wish to apply all or part of the findings elsewhere [21].

## Results

### Participant characteristics

A total of 40 participants across four hospitals were interviewed. No participants dropped out of the study. On average, participants were 41.5 (SD: 9.7) years of age and employed within their hospital for 10.7 years (SD: 7.4). Diversity of roles was represented, although the majority of participants were nurses (See Table 2).

**Table 2:** Participant Characteristics

	**Site A**	**Site B**	**Site C**	**Site D**	**Total participants**
Mean age (SD)	44.1 (12.4)	43.0 (10.9)	45.4 (8.3)	36.9 (8.6)	41.5 (9.7)
Mean years at hospital (SD)	10.4 (6.1)	10.9 (7.8)	13.9 (10.2)	9.6 (7.5)	10.7 (7.4)
Role					
Physician	2	0	2	3	7
Nurse	3	4	2	4	13
Site project leader	2	2	2	2	8
Senior hospital leader	0	2	1	1	4
Case manager	1	1	2	1	5
Other	0	2	1	0	3

### Themes

We identified three overarching themes from the analysis: (1) COVID exacerbated existing problems and created new ones, (2) RESET and quality improvement overall were not the priority during the pandemic, and (3) RESET implementation fidelity regressed because of COVID. We structure the presentation of our findings around these themes and present quotes to illustrate the findings.

#### Theme 1: COVID exacerbated existing problems and created new ones

Sites initially experienced lower patient volumes than anticipated, intensifying pre-existing financial pressures. When patient volume subsequently increased, patients were sicker, but staffing was limited due to pre-existing nurse shortages and the need for exposed and sick staff members to stay at home. Collectively, these actions created new problems including changes in workload, workflow, communication, and worsened morale.

##### Finances and patient volume

Hospitals initially experienced lower patient volume than anticipated as elective surgical procedures were postponed and patients without COVID avoided coming to the hospital. The lower patient volume exacerbated pre-existing financial pressures. To offset the loss of revenue, hospitals instituted furloughs, decreased compensation, and requested staff to take vacations earlier than planned.

Early in that March to May [2020] timeframe, we did not really see the volume, I think we might have had maybe 13 patients total at a time. It wasn't until the summer, late June to early July [2020], where we saw a pretty significant spike [in patient volume]. (Participant 12, Nurse, Site B)

Because when you don't have a census, you can't have seven physicians rounding on those 60 patients. I mean that's something (instituting a furlough) we had to do. ... I mean, initially there were a lot of grudges about [the furloughs]. Nobody likes a pay cut, right? (Participant 4, Physician, Site A)

However, when patient volume subsequently increased, patients were sicker because they had delayed coming to the hospital earlier in their course of illness to avoid exposure to the COVID virus.

The patients that are coming are so, so sick, because everybody's doing everything they can to avoid going to the hospital. By the time we get them, they're a lot sicker than they probably were if we weren't in COVID. (Participant 18, Nurse, Site B)

##### Staffing

Staffing became a major challenge as patient volume increased, especially because of a pre-existing nursing shortage. Although the RESET sites represented four vastly different regions of the U.S., there was a nursing shortage in all of them.

Going into COVID, we had a significant amount of vacancies [for nurses]. Our area that we're in, it's not a very growing town…not a lot of people tend to want to come to [our small town] to live and work. (Participant 23, Nurse Site Leader, Site C)

COVID further exacerbated the nursing shortage because community spread of COVID affected healthcare workers too. With fewer nurses available to care for patients, hospitals had to restrict or “cap” the number of beds available for use.

We had quite a few folks out on quarantine as well with a fairly high positivity rate with those folks out on quarantine. I think throughout the last three months, we probably had to cap some units purely based on not having nursing. (Participant 22, Physician Senior Leader, Site C)

##### Precautions

COVID created new problems because of precautions that had to be taken to prevent infection spread such as the use of personal protective equipment and social distancing. These precautions led to changes in workload, workflow, and communication.

COVID, in general, changed our nursing workflow entirely. It changed the way that we assign patients. Multiple times, we changed who cared for these patients. The additional roles that were created to care for these patients, such as a personal protective equipment observer. So, we had staff that would just observe and make sure that everybody was putting their equipment on the correct way. (Participant 5, Nurse, Site A)

We have nurses who get stuck in isolation rooms because we're the COVID unit, which means we're gowned up and all the gear, we don't have our phones with us. You can't get ahold of us. I think there's definitely been some lack of communication there. (Participant 39, Nurse, Site D)

##### Emotional states

As a result of both pre-existing and new problems brought about by COVID, morale was low, and anxiety and stress were high.

Everybody is more stressed out and management is stressed out, but they're trying so hard to be supportive. People's cups are a little bit fuller in the not so good way...and increased patient acuity and those go hand in hand. We have the employee assistance program. They always are trying to show you, "Here's your extra resources. Here's what we can do for you." But at the end of the day, it is just your workplace, and they can't manage all of your mental health needs and stuff. (Participant 32, Nurse, Site D)

#### Theme 2: RESET, and quality improvement overall, were not the priority during the pandemic

Participants described going into survival mode. As a result, quality improvement efforts, like RESET, fell to the wayside.

##### Survival mode

RESET and quality improvement were not priorities as hospital sites dealt with the challenges presented by the pandemic. Participants described going into survival mode, focusing on planning, and hoping to make it through the most immediate challenges.

Just that with COVID being new and us learning about COVID, I think that we were...in a survival mode for so long. [And] there were lots of different areas that we kind of let fall to the wayside. (Participant 6, Nurse, Site A)

Pre-existing quality improvement efforts like RESET, and others as well fell to the wayside. For example, one site developed a new discharge order set, but implementation did not go well because of the pandemic.

Being in the pandemic, some of these [discharge order sets] went by the wayside and were not really utilized as well as they could have. We have that data to show it just wasn't being used, sadly. (Participant 17, Case Manager, Site B)

##### New priorities

Rather than maintaining emphasis on QI projects, leaders instead focused on supporting staff amidst COVID challenges. Higher-level leadership recognized the value of their physical presence as a sign of support to staff and also to front line management. For the staff, higher-level leaders could provide information beyond that immediately available to front line management. Moreover, existing QI meetings, like those for RESET, were cancelled as leaders recognized that professionals did not have capacity to do anything other than day-to-day tasks because of COVID.

From the director level, we’ve kind of changed the way we’ve done things... we’ve organized a rounding schedule where we go to the units and hit their huddles and just field any questions that they have from a higher-level perspective than their frontline managers might be able to provide. And just trying to be a little bit more present and supportive to them. (Participant 23, Nurse Site Leader, Site C)

I think [COVID] has affected our RESET planning because we haven’t had the meetings we used to have because people just don’t have the bandwidth to do anything extra right now. (Participant 38, Physician, Site D)

##### Excuse to abandon RESET

Notably, COVID was used by some healthcare professionals as an excuse to abandon RESET. Detractors were quick to stop RESET interventions, specifically efforts to localize physicians and attend interprofessional rounds. Leaders seemed too distracted with the COVID response to be able to counter these efforts or plan adaptations to RESET that might preserve fidelity and leverage the interventions to improve the hospital’s COVID response.

We don’t need six people in a room rounding on patients during COVID. When this hit, we weren’t really seeing many COVID patients, but it was scary. I think [detractors] used it as a really good excuse to be like, “Nope, we’re not bedside rounding anymore.” So, I think people who felt really strongly about [not supporting RESET] were really ready when COVID came along to jump off and not be supportive of it. (Participant 32, Nurse, Site D)

#### Theme 3: RESET implementation fidelity regressed because of COVID

Fluctuating patient census, staffing shortages, and isolation precautions contributed to the decline in implementation fidelity. Sites varied in their ability to renew implementation efforts as COVID waves subsided.

##### Fidelity of RESET regressed

As a result of pre-existing and new problems created by the COVID pandemic and lack of prioritization of QI initiatives, the fidelity of all five RESET interventions regressed during COVID. For example, sites found it difficult to localize physicians to specific units due to fluctuating patient census, increased internal transfers, and staffing shortages. Interprofessional rounds regressed in location, attendance, and format due to social distancing. Some sites stopped interprofessional rounds altogether for a period of time. The sites which had been doing interprofessional rounds at the bedside moved these rounds to a conference room to improve social distancing, but the capacity of conference rooms was restricted so that not all professions were represented. In addition, attendance by case managers and social workers declined as many began to work from home.

It turned out that we just couldn't meet in the room together because there was too many of us, because there were usually 6 or 7 nurses trying to report, so we couldn’t be in the room waiting and we couldn’t have extra people come to the meeting because that increased our numbers. (Participant 13, Nurse, Site B)

The fidelity of other RESET interventions also regressed. Unit Nurse-Physician Co-leadership was a RESET intervention that required the unit nurse leader and the physician leader assigned to the unit to meet regularly and jointly lead quality improvement activities. However, as COVID cases increased, the need for physician unit leaders to provide patient care grew and unit nurse leaders prioritized COVID response efforts over other quality improvement efforts with the result that co-leadership stopped.

[Co-leadership], more than anything else, has probably fallen to the wayside. (Participant 10, Physician Senior Leader, Site B)

Patient engagement efforts, another RESET intervention, included the use of whiteboards in patient rooms to define goals and the daily plan of care. However, the use of whiteboards diminished because, without bedside interprofessional rounds, professionals entered patient rooms less frequently as a team, resulting in less accountability to ensure whiteboards in patient rooms were complete and accurate.

I would always write my name and phone number up there [on the whiteboard] and an anticipated discharge date. And since I haven't been going into the COVID rooms, I've not done that. (Participant 27, Case Manager, Site C)

##### Variation in renewed implementation of RESET

Sites varied in their ability to renew their implementation efforts as COVID waves subsided. One site, which had changed from unit-based interprofessional rounds to a larger hospital-wide interprofessional rounds meeting, had no immediate plans to return to pre-COVID processes. Another site temporarily moved interprofessional rounds from the bedside to a conference room and reinstated bedside interprofessional rounds after several months. When interprofessional rounds did resume, some sites struggled to reengage physicians who either were not on staff prior to COVID or did not remember their role in RESET.

Our system level [leaders] mentioned in-room rounding, [stating] that it would be best practice [to do bedside rounds] as long as we can follow the social distancing guidelines. (Participant 23, Nurse Site Leader, Site C)

So, we eventually made the decision to stop RESET based on the COVID precautions. When we made the decision to restart…we had multiple encounters with physicians who didn't understand RESET. (Participant 5, Nurse, Site A)

## Discussion

In this study of an ongoing quality improvement effort at 4 hospitals, we found that COVID exacerbated existing problems and created new ones that led to changes in workload, workflow, communication, and emotional states. These problems resulted in diversion of attention and effort away from quality improvement efforts, like RESET. Sites varied in their ability to resume implementation efforts as COVID waves subsided.

COVID led to complex changes in our study sites yielding multiple downstream effects. Hospitals initially experienced lower than anticipated patient volumes, resulting in exacerbation of financial pressures. When patient volume did increase, sites struggled to staff available beds, due to pre-existing nurse staffing shortages and the need for exposed and sick staff members to stay at home for up to two weeks. Our findings corroborate prior research showing initial reductions in overall hospital volume in many parts of the U.S. as patients with conditions other than COVID avoided emergency departments and hospitals cancelled nonurgent surgeries [22–24]. The postponement of more lucrative procedural services and the mismatch between supply and demand on hospital resources created financial strain for many U.S. hospitals with estimates that more than a third would maintain a negative operating margin through 2021 [25]. Hospitals in our study initially dealt with financial pressure by instituting furloughs and requesting staff to take vacations earlier than planned, but this approach seemed to only worsen the psychological stress experienced by healthcare professionals. These findings are also consistent with prior research showing high levels of stress among professionals in acute care settings during the pandemic, especially when they did not feel valued by their employers [26–28]. Hospitals in our study experienced high nursing turnover and used travel nurses to fill staffing shortages, similar to many U.S. hospitals [29].

Professionals in our study experienced changes to both workload as COVID volumes fluctuated, and workflows as increased precautions were implemented. Hospitals created designated space for COVID patients, changed policies to enforce social distancing, and established processes for personal protective equipment (PPE) usage. These workflow changes led to communication challenges. PPE served as a physical barrier to communication. Social distancing practices also served as a barrier to communication, leading to a regression in interprofessional rounds and reducing the opportunity for teams to meet in person to talk about their patients. Prior research similarly showed that material barriers (i.e., PPE) and spatial barriers (i.e., social distancing) implemented during the pandemic disrupted workflow and communication [30]. Notably, hospitals varied in their ability to reinstate interprofessional rounds as COVID waves subsided. The decrease in formal structures to facilitate collaboration is concerning since literature shows teamwork can improve outcomes and help with nurse retention [31, 32].

Our study describes how hospital leaders’ attention and effort were diverted from an ongoing quality improvement initiative because of the pandemic. At the start of the pandemic, experts called for the use of quality improvement methods and implementation science in healthcare organizations’ efforts to manage challenges posed by the pandemic [1–4]. Studies describe the rapid adjustments hospitals made to staffing, space, and how services were provided to patients [27, 33–35], but we know little about how the pandemic affected ongoing quality improvement efforts. Our study helps explain why quality and safety performance appears to have worsened during the pandemic [6, 7, 36]. Participants in our study described going into survival mode and focusing only on the most immediate challenges related to the pandemic. Quality improvement efforts, like RESET, fell to the wayside. Furthermore, detractors used COVID to abandon ongoing quality improvement efforts. Our findings support concerns raised by others that hospitals were significantly strained throughout the pandemic [37], which led healthcare organizations to pull resources and attention away from traditional programs for quality and patient safety [5, 38].

Our findings also support the need for research to identify elements important for fostering hospital resilience. Some RESET sites were able to resume implementation efforts with relative ease as COVID waves subsided, while others struggled. Traditional approaches to understanding hospitals’ response to the pandemic focus on the “4 s” framework of preparedness: staff, stuff, space, and systems [39, 40]. This framework emphasizes the need for adequate staffing levels, sufficient space for anticipated patient volume, adequate supplies of essential equipment (e.g., PPE), and systems to manage these resources. It may be more useful to evaluate hospitals’ experience through the lens of organizational resilience. Sutcliffe and Vogus describe organization resilience as the power of organizational units to resume, rebound, bounce back, or positively adjust when faced with untoward events [41]. A hospital exhibiting organizational resilience during the pandemic would ensure the delivery of high-quality care for patients hospitalized with COVID and while also maintaining continuity of operations to ensure high-quality care for non-COVID patients. Though others have also reported variation in hospitals’ ability to provide high-quality care to non-COVID patients during the pandemic, further research is needed to confirm this variation and identify elements most important to fostering hospital resilience and ensuring high-quality care during crises [42].

### Limitations

Our study has several limitations. First, we evaluated the effect of COVID on a quality improvement project that aimed to redesign microsystems of care with a fairly complex and multifaceted intervention. Though we believe our findings likely reveal factors affecting other types of quality improvement efforts, it is possible that other, less complex efforts were affected differently during COVID. Second, our interviews spanned over seven months (November 2020 through May 2021). As the pandemic continued, it is possible that some hospitals learned to mitigate the disruption the pandemic had on long term quality improvement efforts. Third, we did not interview every healthcare professional who was part of RESET. We used purposive sampling to include a range of participants and achieved code and meaning saturation. Therefore, we feel that our findings are representative of the hospitals’ experience with advancing quality during the pandemic.

## Conclusion

We found that COVID exacerbated existing problems and created new ones that led to changes in workload, workflow, communication, and emotional states. These problems resulted in a diversion of attention and effort away from quality improvement efforts, like RESET, and regression in fidelity of implementation. Our findings are important in explaining how COVID adversely affected hospitals’ non-COVID quality improvement efforts throughout the pandemic. Future research should identify elements important for fostering hospital resilience and ensuring high-quality care during crises.

## Supplementary Information

**Additional file 1** .

## Data Availability

The datasets used and/or analyzed during the current study are available from the corresponding author on reasonable request due to privacy of our interviewees.

## Abbreviations

RESET: ***RE*** *designing* ***S*** *yst* ***E*** *ms to Improve* ***T*** *eamwork and Quality for Hospitalized Patients*
COVID: SARS-CoV-2/Covid-19
AIMS: Advanced and Integrated Microsystems
COREQ: Consolidated Criteria for Reporting Qualitative Research
PPE: Personal protective equipment

## References

[1] Hirschhorn L, Smith JD, Frisch MF, Binagwaho A (2020). Integrating implementation science into covid-19 response and recovery. BMJ.

[2] Ovretveit J, Mittman BS, Rubenstein LV, Ganz DA (2021). Combining Improvement and Implementation Sciences and Practices for the Post COVID-19 Era. J Gen Intern Med.

[3] Shah A, Pereira P, Tuma P (2021). Quality improvement at times of crisis. BMJ.

[4] Wensing M, Sales A, Armstrong R, Wilson P (2020). Implementation science in times of Covid-19. Implement Sci.

[5] NPSD Data Spotlight (2021). Patient Safety and COVID-19: A Qualitative Analysis of Concerns During the Public Health Emergency.

[6] Weiner-Lastinger LM, Pattabiraman V, Konnor RY, Patel PR, Wong E, Xu SY, Smith B, Edwards JR, Dudeck MA (2022). The impact of coronavirus disease 2019 (COVID-19) on healthcare-associated infections in 2020: A summary of data reported to the National Healthcare Safety Network. Infect Control Hosp Epidemiol.

[7] Baker MA, Sands KE, Huang SS, Kleinman K, Septimus EJ, Varma N, Blanchard J, Poland RE, Coady MH, Yokoe DS (2021). The Impact of COVID-19 on Healthcare-Associated Infections. Clin Infect Dis..

[8] Fakih MG, Bufalino A, Sturm L, Huang R-H, Ottenbacher A, Saake K, Winegar A, Fogel R, Cacchione J (2022). Coronavirus disease 2019 (COVID-19) pandemic, central-line-associated bloodstream infection (CLABSI), and catheter-associated urinary tract infection (CAUTI): The urgent need to refocus on hardwiring prevention efforts. Infect Control Hosp Epidemiol.

[9] CMS Announces Relief for Clinicians (2020). Providers, Hospitals and Facilities Participating in Quality Reporting Programs in Response to COVID-19.

[10] FY 2023 Hospital Inpatient Prospective Payment System (IPPS) and Long Term Care Hospitals (LCTH PPS) Propose Rule - CMS-1771-P [https://www.cms.gov/newsroom/fact-sheets/fy-2023-hospital-inpatient-prospective-payment-system-ipps-and-long-term-care-hospitals-ltch-pps].

[11] O'Leary KJ, Johnson JK, Manojlovich M, Goldstein JD, Lee J, Williams MV (2019). Redesigning systems to improve teamwork and quality for hospitalized patients (RESET): study protocol evaluating the effect of mentored implementation to redesign clinical microsystems. BMC Health Serv Res.

[12] Redesigning Systems to Improve Teamwork and Quality for Hospitalized Patients (RESET Project) [https://www.ahrq.gov/patient-safety/settings/hospital/resetguide.html]. 10.1186/s12913-019-4116-z PMC6505207 31068161

[13] Tong A, Sainsbury P, Craig J (2007). Consolidated criteria for reporting qualitative research (COREQ): a 32-item checklist for interviews and focus groups. Int J Qual Health Care.

[14] Campbell S, Greenwood M, Prior S, Shearer T, Walkem K, Young S, Bywaters D, Walker K (2020). Purposive sampling: complex or simple?. Res case examples J Res Nurs.

[15] VERBI Software (2019). MAXQDA 2020.

[16] Hsieh H-F, Shannon SE (2005). Three Approaches to Qualitative Content Analysis. Qual Health Res.

[17] Miles MB, Huberman AM, Saldaña J (2014). Qualitative data analysis: A methods sourcebook. 3rd.

[18] Hennink MM, Kaiser BN, Weber MB (2019). What Influences Saturation? Estimating Sample Sizes in Focus Group Research. Qual Health Res.

[19] Golafshani N (2003). Understanding reliability and validity in qualitative research. Qual Rep.

[20] Cypress BS (2017). Rigor or Reliability and Validity in Qualitative Research: Perspectives, Strategies, Reconceptualization, and Recommendations. Dimens Crit Care Nurs.

[21] Lincoln YS, Guba EG (1986). But is it rigorous? Trustworthiness and authenticity in naturalistic evaluation. New directions for program evaluation.

[22] Blecker S, Jones SA, Petrilli CM, Admon AJ, Weerahandi H, Francois F, Horwitz LI (2021). Hospitalizations for Chronic Disease and Acute Conditions in the Time of COVID-19. JAMA Intern Med.

[23] Khan SS, Furmanchuk A, Seegmiller LE, Ahmad FS, Black BS, O'Leary KJ (2021). Divergent Trends in Emergency Department Presentations Amid the Novel Coronavirus Disease 2019 Pandemic in Chicago. Illinois J Card Fail.

[24] Findling MG, Blendon RJ, Benson JM (2020). Delayed Care with Harmful Health Consequences—Reported Experiences from National Surveys During Coronavirus Disease 2019. JAMA Health Forum.

[25] Kaufman Hall (2021). Financial Effects of COVID-19: Hospital Outlook for the Remainder of 2021.

[26] Prasad K, McLoughlin C, Stillman M, Poplau S, Goelz E, Taylor S, Nankivil N, Brown R, Linzer M, Cappelucci K et al: Prevalence and correlates of stress and burnout among U.S. healthcare workers during the COVID-19 pandemic: A national cross-sectional survey study. EClinicalMedicine 2021,35:100879. 10.1016/j.eclinm.2021.100879 PMC8141518 34041456

[27] Turner S, Botero-Tovar N, Herrera MA, Borda Kuhlmann JP, Ortiz F, Ramírez JC, Maldonado LF (2021). Systematic review of experiences and perceptions of key actors and organisations at multiple levels within health systems internationally in responding to COVID-19. Implement Sci.

[28] Heidarijamebozorgi M, Jafari H, Sadeghi R, Sheikhbardsiri H, Kargar M, Gharaghani M (2021). The prevalence of depression, anxiety, and stress among nurses during the coronavirus disease 2019: A comparison between nurses in the frontline and the second line of care delivery. Nurs Midwifery Stud.

[29] Yang YT, Mason DJ. COVID-19's impact on nursing shortages, the rise of travel nurses, and price gouging. Health Affairs Forefront. 2022. https://www.healthaffairs.org/do/10.1377/forefront.20220125.695159. Accessed 1 June 2022.

[30] Hayirli TC, Stark N, Bhanja A, Hardy J, Peabody CR, Kerrissey MJ (2021). Masked and distanced: a qualitative study of how personal protective equipment and distancing affect teamwork in emergency care. Int J Qual Health Care..

[31] Mohr DC, Burgess JF, Young GJ (2008). The influence of teamwork culture on physician and nurse resignation rates in hospitals. Health Serv Manage Res.

[32] Blakeney EA, Chu F, White AA, Smith GR, Jr., Woodward K, Lavallee DC, Salas RME, Beaird G, Willgerodt MA, Dang D et al: A scoping review of new implementations of interprofessional bedside rounding models to improve teamwork, care, and outcomes in hospitals. J Interprof Care 2021:1–16. 10.1080/13561820.2021.1980379 PMC8994791 34632913

[33] Auerbach A, O'Leary KJ, Greysen SR, Harrison JD, Kripalani S, Ruhnke GW, Vasilevskis EE, Maselli J, Fang MC, Herzig SJ (2020). Hospital Ward Adaptation During the COVID-19 Pandemic: A National Survey of Academic Medical Centers. J Hosp Med.

[34] Kumar SI, Borok Z: Filling the Bench: Faculty Surge Deployment in Response to the Covid-19 Pandemic. NEJM Catalyst 2020.

[35] Linker AS, Kulkarni SA, Astik GJ, Keniston A, Sakumoto M, Eid SM, Burden M, Leykum LK (2021). Bracing for the Wave: a Multi-Institutional Survey Analysis of Inpatient Workforce Adaptations in the First Phase of COVID-19. J Gen Intern Med.

[36] Fakih MG, Ottenbacher A, Yehia B, Fogel R, Miller C, Winegar A, Jesser C, Cacchione J (2022). COVID-19 hospital prevalence as a risk factor for mortality: an observational study of a multistate cohort of 62 hospitals. BMJ Qual Saf.

[37] Grimm CA (2021). Hospitals Reported that the COVID-19 Pandemic has Significantly Strained Health Care Delivery.

[38] Fleisher LA, Schreiber M, Cardo D, Srinivasan A (2022). Health Care Safety during the Pandemic and Beyond — Building a System That Ensures Resilience. N Engl J Med.

[39] Fiest KM, Krewulak KD (2021). Space, Staff, Stuff, and System: Keys to ICU Care Organization During the COVID-19 Pandemic. Chest.

[40] Uppal A, Silvestri DM, Siegler M, Natsui S, Boudourakis L, Salway RJ, Parikh M, Agoritsas K, Cho HJ, Gulati R (2020). Critical Care And Emergency Department Response At The Epicenter Of The COVID-19 Pandemic. Health Aff (Millwood).

[41] Sutcliffe KM, Vogus TJ: Organizing for resilience. In: Positive Organizational Scholarship: Foundations of a New Discipline. edn. Edited by Cameron KS, Dutton JE, Quinn RE. San Francisco, CA: Berrett-Koehler; 2003:94–110.

[42] Barbash IJ, Kahn JM (2021). Fostering Hospital Resilience—Lessons From COVID-19. JAMA.

