# Supplementary material for: Effect of COVID-19 on the implementation of a multifaceted intervention to improve teamwork and quality for hospitalized patients: a qualitative interview study
Source: BMC Health Serv Res. 2022 Nov 19;22:1379. doi: 10.1186/s12913-022-08795-5 (PMC9675968; doi:10.1186/s12913-022-08795-5)
Supplement: Supplementary file 1 — Additional file 1 . [file 12913_2022_8795_MOESM1_ESM.pdf]

Interview guide

Topics-to-learn about:

Topic 1: COVID-19's effect on Hospital
Topic 2: COVID-19's influence on RESET
Topic 3: Feedback's influence on implementation of RESET
Topic 4: RESET influence on COVID response

Start: Thank you so much for taking the time to talk to me about COVID and RESET today. To start off I want to first understand how COVID has affected your hospital.

Topic 1: COVID-19's effect on Hospital

1. Sites have differed in both the volume of COVID-19 patients seen and the timing of COVID-19 volume. Some information on your experience will help us put our discussion in perspective. Tell us about COVID-19 patient volume that your hospital has experienced.
   - o When did COVID-19 volume peak? / When do you expect COVID-19 volume to peak?
   - o What does the trend look like now?

2. Tell us about how your non-COVID patient volume has been affected during the pandemic.
   - o What has been the effect of the change in non-COVID patient volume?
   - o What does the trend look like now?

3. Tell us about your hospital's and health system's response to these changes in patient volumes?
   - o How will/has/did staffing change (nurse, physician, social work, pharmacist, etc)?
   - o How will/has/did space or units change?
   - o How will/were changes communicated and operationalized?
   - o How prepared was your hospital for change?

Explicit transition: *Ok, great... now that you told me about how COVID has affected your hospital, I am curious about any effect of COVID on RESET.*

Topic 2: COVID-19's influence on RESET

1. How has COVID-19 influenced the implementation of RESET interventions?

- o In what ways has COVID-19 gotten in the way of implementation?
- o In what ways has COVID-19 improved implementation?
- o How has COVID-19 influenced the implementation of…
    - Unit-Based Physician Teams (localization of physicians)?
    - Unit Nurse-Physician Co-leadership?
    - Enhanced Interprofessional Rounds?
    - Unit-level Performance Reports?
    - Patient Engagement Activities?

2. What kinds of changes or alterations did you think you will need to make to RESET so it will work effectively in your setting during COVID?
    - o Who will decide (or what is the process for deciding) whether changes are needed to RESET so that it works during COVID?

3. What has allowed you to continue to make progress with RESET implementation during the COVID-19 pandemic?
    - o What has been especially helpful in navigating challenges related to COVID-19?

4. What challenges have you experienced in making progress with RESET implementation during the COVID-19 pandemic?
    - o What has gotten in the way of navigating challenges related to COVID-19?

Explicit transition: *Great. Now I am going to ask about feedback during RESET*

Topic 3: Feedback's influence on implementation of RESET

1. What supports have been the most helpful to you as you implement and use the intervention?
    - Online resources
    - RESET guide
    - Mentor meetings
    - Leaders

2. What kind of information exchange do you have with others outside your setting related to RESET?
    - Site webinars
    - Mentor meetings
    - Other site

3. When you need to get something done or to solve a problem related to RESET, who are your "go-to" people?

Explicit transition: *Great. Now I want to hear your thoughts about how RESET has influenced your hospital's or unit's response to COVID.*

Topic 4: RESET influence on COVID response

1. How has the RESET project influenced your hospital's response to changing patient volumes related to COVID-19?
   - o How did working as a RESET team influence your ability to respond?
   - o How did the RESET interventions influence your ability to respond?

2. To what extent would implementing RESET provide an advantage for your organization compared to other organizations in your area during COVID?

***Explicit transition*:** I really appreciate you taking the time to talk with me today.

Conclusion

1. Is there anything about RESET or COVID-19 that we did not talk about today that you think we should know?

Closing Remarks: Thank you again for sharing your experience. Once we have finished the interviews and analyzed the data, we will share our findings with you to get your thoughts on whether or not we got it right.
